# Caring for a cerebral palsy child: a caregivers perspective at the University Teaching Hospital, Zambia

**DOI:** 10.1186/s13104-017-3011-0

**Published:** 2017-12-08

**Authors:** Brian Chanda Chiluba, Geoffrey Moyo

**Affiliations:** Department of Physiotherapy, School of Health Sciences, The University of Zambia, Lusaka, Zambia

**Keywords:** Experiences, Parent/caregivers, Cerebral palsy, Modified caregiver strain index (MCSI)

## Abstract

**Background:**

Cerebral palsy is a major cause of disability and most survivors are left with residual disability and are dependent on parents/caregivers for essential care. This study aimed to determine the experiences of parents/caregivers of cerebral palsy children receiving out-patient physiotherapy. A concurrent mixed methods was used to collect data in the present study. The modified caregiver strain index (MCSI-13) was used to detect Disturbed sleep, Inconvenient/Tiresome, Physical strain, Confining, Family changes, Changes in personal plan, Other demands, Emotional adjustments, Upsetting behavior, Patient has changed, Work adjustments, Financial Strain and Feeling Overwhelmed (strain morbidity) in 25 parents/caregivers of CP children. A purposive sample of 25 parents/caregivers was selected for both the quantitative part and qualitative part of the study. The study was conducted at Community Based Intervention Association Out-patients at the University Teaching Hospital in Lusaka, Zambia. The MCSI was used to collect quantitative data and in-depth interviews provided the qualitative data.

**Results:**

The median age of the participants was 33.6 years and a range of 27 to 50 years. The study sample consisted of more females (92%) than males (8%). being overwhelmed and inconvenient/tiresome followed by family adjustments and work adjustment 72 and 68% respectively for each were the experiences mostly highlighted by the parents/caregivers in this study. When it came to the needs of the parents/caregivers more than half of them needed help with caring. To this effect participants expressed their perception; one mother had this to say, *“…I need someone to help in caring. Sometimes I need to do some other things but can’t, because if I do then no one will remain with the child…”.*

**Conclusion:**

This study point out to some evidence that the burden inflicted on those caring for children with cerebral palsy should be addressed if the quality of care for those with cerebral palsy is to be improved.

## Background

Parents/caregivers of children with Cerebral Palsy (CP) often shoulder the principal multifaceted responsibilities of long-term CP and disability care. CP is one such developmental disorder that begins in early childhood as a set of functional limitations that stem from disorders of the developing central nervous system [1]. It is estimated that worldwide up to 9.3% of youths under the age of 18 have a condition that can be classified as neurodevelopmental such as CP, epilepsy and developmental delay. The current estimated incidence of CP is 2–3 per 1000 live births in developed countries. However, it is difficult to establish the incidence of cerebral palsy in most developing countries because CP is not captured in the population census or any other surveys in most developing countries, though the incidence is higher in males than in females [1, 2].

**Table 1:** Educational status, employment status and income level of caregiver (n = 25)

Variable	Frequency	Percent
Education		
College/university	13	52.0
Grade 12	8	32.0
Grade 9	3	12.0
Grade 7	1	4.0
Never been to school	0	0.0
Employment status		
Employed	9	36.0
Unemployed	10	40.0
Self employed	6	24.0
Income level		
No income	10	40.0
K1000 above	6	24.0
K510–K900	5	20.0
K250–K500	3	12.0
Below 250	1	4.0

Illustrated in Table 1 above are employment status, educational status and the monthly income levels of parents/caregivers. The majority of parents/caregivers had a College/University education level. Those that neither reached Grade 12 nor went to school accounted to 48% of the population. Only 52% of the parents/caregivers had obtained a college/university education. The results of the present study revealed that the majority (40%) of the parents/caregivers were unemployed and having no income at all during the time of data collection. A total of 36% had to care for their child with cerebral palsy while having a full time job

**Table 2:** Modified caregiver’s strain index scores (n = 25)

MCSI scores	Frequency	Percent
0–6	9	36
7–9	16	64
10–13	0	0

Caregiver strain was measured using the 13-item modified caregiver strain index. Every “yes” response was recorded as 1 and the sum of “yes” responses was scored. Scores of 7 and more on the scale indicated high level of strain. The mean MCSI-13 score was 7.48 (SD = 3.293, range 8–10). The majority of the caregivers reported a higher strain level with 64% scoring 7 and above on the scale. Table 2 above presents the frequencies of scores on the MCSI

In India 4 million people are affected by CP annually [1]. Parents/caregivers of CP children play a central role in the lives of these children and therefore their own well-being is inextricably linked to that of the children. Two major social trends make the consideration of parents/caregivers health particularly relevant. Firstly, there has been a decided shift towards Community Based Rehabilitation (CBR) of children with disabilities. In addition to maximizing opportunities for social inclusion, Community Based Rehabilitation recognizes parents/caregivers as partners in the provision of care. Secondly, in some developed nations the principles of family-centered care have been embedded in health policy and embraced by health and social services agencies providing services to children with disabilities and their families. Parents/caregivers are considered instrumental partners in health policy by influencing the nature and direction of the care their child receives. These trends place greater emphasis on parent/caregiver voice and involvement, and also represent extraordinary parental responsibility [2]. Evidence is mounting regarding the extent to which parents/caregivers of children with neurodevelopmental disorders experience elevated levels of physical and psychological distress [3].

Culturally, in an African perspective and Zambia included, it is expected that a relative will take up the responsibilities of care-giving for the person with a disability or cerebral palsy. The parent/caregivers frequently accept or are expected to assume their role without regard for the possible emotional, physical, and financial consequences [4]. As cerebral palsy is a sudden event, parents/caregivers of CP children are forced to accept a large amount of unforeseen responsibilities in the absence of preparation. In addition to this, the care-giving role has various other implications for the parents/caregivers. These includes future plans being shattered, present income generating activities being abandoned, a decrease in leisure time and susceptibility to a deteriorating health status [5–7]. In the process of care-giving, the abilities of the parents/caregivers to provide for their own emotional, personal, physical, social and financial needs are seriously compromised [8, 9]. According to Bakas [10] needs of parents/caregivers includes general information about the warning signs for cerebral palsy, lifestyle changes for the child with CP, and the management of cerebral palsy related symptoms and complications. To date we know of no study in Zambia that has focused on the impact of caring for children with CP, though a similar study with focus on short-term care of a child with Malaria (The Disability Burden of Malaria in Lusaka urban) was conducted by Chalwe et al., [11] at The University Teaching Hospital (UTH). Thus, the purpose of the present study was to examine the challenges, needs and experiences that parents/caregivers of children with CP face. CP was chosen as a prototype condition to ascertain the needs and challenges of parents/caregivers of children with disabilities.

## Methods

### Study design

The study was mixed methods using a concurrent parallel approach. The quantitative part utilised a non-intervention, cross-sectional descriptive study design with a questionnaire containing closed items. The qualitative part utilised a phenomenological approach with open ended items, these methods that were employed in the study attempted to gain a broader understanding of the caregivers’ perspective when caring for a child with CP. The objective of this research was to parallel a quantitative and a qualitative bus transit research study and to analyze the results in terms of integrating the two methods by use of triangulation.

### Study area

This study was carried out at the UTH, Department of Paediatrics, Community Based Intervention Association (CBIA) formerly known as Action for Disability and Development (ADD). The employees of the Department includes; Physiotherapists, Speech Therapists, administrators and other support staff. The center was instituted to rehabilitate children with disabilities the most common being CP and other related conditions. UTH is located in the capital in Lusaka and it is the biggest referral hospital in the Zambia offering health services at tertiary level. The hospital occupies approximately eight hectares of land spread over one and a half kilometres.

### Study population, sampling and sample selection technique

The study population constituted parents/caregivers of CP children, receiving treatment at the UTH/(CBIA). Though a bigger sample size would have been attained from this study, however, due to limited time in which to carry on a research as prescribed by the academic calendar in which to carry out an academic research by the University, only a smaller sample size was realised for the study in order to carry out the study within the required time. Therefore, a purposive sample of 25 participants was picked from the population of the study for the quantitative study and 5 participants were picked for a qualitative study. The qualitative sample helped in complementing the inefficiencies in inferences for a small quantitative sample by gaining a broader perspective of the phenomenon under study.

### Data collection tools

An interview questionnaire was adapted by the researcher from validated questions from literature to gather social demographic information of parents/caregivers. The modified caregiver strain index (MCSI) was used which is an already validated tool that is used to measure strain. The MCSI is an ordinal scale, used to identify families with potential care-giving strain. It is a 13-question tool that measures strain related to care provision in providing various degrees of care to patients at home. Each item is answered with a “yes”, or “no” responses. Scoring is accomplished through adding all affirmative responses to arrive at a total score; thus, a higher score, 7 or higher than 7 positive responses (yes responses) implies a high level of burden [12]. There is at least one item for each of the following major domains: Employment, Financial, Physical, Social and Time. According to Blake [13] internal consistency reliability is high (alpha = 0.86) and construct validity is supported by correlations with the physical and emotional health of the caregiver and with subjective views of the care-giving situation. The Rehabilitation and Support Questionnaire addresses the caregiver’s perception of rehabilitation and support through five questions. The questions concern the parents’/caregivers’ perceived importance of physiotherapy resulting in different options and the need of support workers for children with cerebral palsy in the community. Other questions address the parents’/caregiver’s rating of rehabilitation, support, information and attention they receive when their child with cerebral palsy is receiving care from the physiotherapists. Even though the above tools have already been validated as they have been used in research, they were subjected to a pilot study for validation before this study.

An eight-item questionnaire was developed from literature specifically for data collection from open-ended items. It contained eight open-ended questions on the needs and experiences of caregivers. To ensure that the interviews were credible and trustworthy, the researcher recorded the interviews verbatim and took field notes. The researcher also ensured that the respondents understood the questions very well. If they were not sure, the questions were repeated and the researcher listened carefully and took field notes.

### Data management and data analysis

For quantitative data analysis, the variables that were used in graphs and charts were as follows; Gender, age, marital status, occupation, education level of the parents, responses to questionnaire included; Gender either male or female, age response ranged from 15 to 90 years, marital status response included single, marriage, divorced and widowed, educational level included secondary and tertiary level and on occupation the following were the responses expected, employed, unemployed and self-employed.

Owing to a small sample size, however, it could be difficult to carry out statistical tests in quantitative research to establish association among variables due to that data may not be normally distributed to allow for parametric tests. Therefore, in order to deal with a smaller sample size in this research with regard to skewed data. Log transformation was done for right skewed data and square transformation was done for left skewed data [14]. This was so in order to assume normality because of skewed data as a result of a small sample and to allow for parametric tests like the ANOVA for association among variables.

The Statistical Package for Social Science (SPSS) version 16 was used to analyze quantitative data relating the results to the contents of the qualitative results. SPSS is a statistical and data management package that can perform a wide variety of statistical procedures including transformation, distribution of data, description, compare groups with significant differences using parametric [15]. Significance level was set at 5%, while descriptive and inferential statistics were employed in analysing data from the closed-ended questionnaire. The results were presented as tables, pie charts and graphs from Microsoft excel. The confidence interval of the difference was at 95%. Data collected using interviews was recorded on a tape recorder. In analyzing qualitative data the researcher transcribed the results that were recorded. Some participants were quoted verbatim. The information was then organized into main themes. Themes for qualitative analysis included; need for support services, work overload, need for community cantered programmes, need for finances, views on physiotherapy rehabilitation services, and need for respite care. Tenets of narrative analysis were used to construct a narrative account of parents’/caregiver’s needs and experiences.

In order to ensure that there was credibility in the quality of the qualitative information data triangulation was done, triangulation was done through multiple analysts and ‘member checks’. And to ensure confirmability codes were categorized according to similar contents and then developed into broader themes. The categories were interpreted in order to determine the real meaning of the text. At the end of the analysis the themes were cross-checked with the interview transcripts to check the validity of the data.

## Results

### Demographic characteristics of respondents

A total of 25 respondents participated in the study of which 8% (n = 2) were males and 92% (n = 23) were females. The median age of respondents was 33.56 with ages ranging from 27 to 50 years.

### Effects and relationships of the demographic variables on the MSCI-13 scores

In order to understand the results of the MSCI scores, it was imperative that factors that could have contributed to the levels of strain other than the perception of the impact of caring for children with cerebral palsy be ascertained.

### Effects of age of respondents on the MSCI scores

The mean MSCI scores of those aged 27–33 years were 7.55 (SD = 3.78), 44–50 years, 7.20 (SD = 2.05). A one-way ANOVA was done to the data and the results showed that the main effect of the age of the respondents was found to be unreliable, F = 0.043, p > 0.05. It can be inferred that the age of the respondents did not affect the levels of strain scored on the MSCI.

### Effects of gender of the parent/caregiver on the MSCI scores

Parents/caregivers who were male and taking care of a cerebral palsy child had a mean MSCI score of 10.5 (SD = 0.71), while those who were female had 7.22 (SD = 3.30). When a one-way ANOVA was done on the data, the main effects of gender were not found to be significant, F = 1.897, p > 0.05. It can thus be concluded that the levels of strain was unrelated to the gender of the caregiver of cerebral palsy.

### Effects of relationship of parent/caregiver with the child on the MSCI scores

The mean MSCI score of parent was 7.60 (SD = 2.72) others, Elder sister/brother 6.25 (SD = 5.189), Friend/neighbor 8.0 (SD = 3.69). However, when a one-way ANOVA was done on the data, the main effects of the relationship with a child with cerebral palsy was found not to be significant, F = 0344, p > 0.05. It can be concluded that the relationship of parent/caregiver with child was unrelated to levels of strain.

### Effects of marital status on the MSCI scores

The mean MSCI scores with regards to marital status is as follows the highest mean score represented those who were married and the lowest score was for the divorced. When a one-way ANOVA was done on the data, the main effects were unreliable suggesting that the effects of status did not affect the levels of psychological distress F = 0.12, p > 0.05.

### Effects of employment status on the MSCI scores

The mean MSCI scores of those formally employed were 7.0 (SD = 2.8), those unemployed, 8.0 (SD = 3.98) and self-employed, 7.33 (SD = 3.98). A one-way ANOVA was done on the data and the results showed that the main effect of employment status on the MSCI scores was found not to be reliable, F = 0.211, p > 0.05. It can be concluded that the employment status had no influence on the levels of strain.

### Effects of educational level on the MSCI scores

The parents/caregivers had more or less similar MSCI scores. The mean score of those who never went to school as far as Grade 7 was 9.0, those who reached GRADE 9 level 5.0 (SD = 2.65) those that reached up to Grade 12 had 8.25 (SD = 2.12) and those that went to the university/college 7.46 (SD = 3.97). The one-way ANOVA was done showed that the main effect of educational level on the MSCI scores was not found to be significant, F = 0.761, p > 0.05. It can be inferred that the educational level did not affect the levels of strain scored on the MSCI.

## Qualitative analysis

To have deeper understanding and meaning of the qualitative results, data was obtained, processed by coding and analysis then presented in a verbatim form under appropriate themes; this followed a classical approach, employing Malterud’s ‘Systematic text condensation’, a descriptive and explorative method for thematic cross-case analysis drawing upon Giorgi’s psychological phenomenological analysis.

### Need for respite care

More than half of the caregivers needed help with caring. They expressed a need for someone to stand in for them to have a chance to do other duties.

A2: *“…I require a person to help me so that I am not alone helping the sick person….”* (Male caregiver: mother)
A5: *“…I need someone to help in caring. Sometimes I need to do some other things but can’t, because if I do then no one will remain with the child…”* (Female caregiver:mother)
A4: *“…The other help I need is for someone to help me in dressing her, bathing her…it is a very big task to bath her and it takes all my time….”* (Female caregiver:mother)

All the interviewed caregivers, but one, were not able to find respite care by employing someone whenever they wanted to go and attend to other matters. One caregiver was able to get respite care by delegation of duties to other members of the family.

A5: *“…My husband and I have decided to continue with our normal activities. What we have done is delegate duties to everyone at home…”* (Female caregiver:mother)
A1: *“…I have someone who does come to be with the child when I need to go out like shopping and sometimes I have to go and see my sick mother. I do pay her. In fact my sister in law helps me to pay the girl….”* (Female caregiver:mother)

### Need for home based care and community support workers

A significantly high percentage of the parents/caregivers (76.0%) responded positively to the importance of having community support workers. Only 20.0% considered it as important and 4.0% perceived it as less important.

Parents/caregivers complained that bringing the patient to the hospital was a very tough thing to do physically and financially. They, for this reason, emphasized that they needed to have community health workers conducting treatment sessions in their homes.

A1: *“…We need community health workers to do visits in our homes than us coming here, because it is very costly….”* (Mother)
A3: *“…If physiotherapists could come to homes it would be better….”* (Female caregiver:mother)
A1: *“…Help is needed especially if the physiotherapists can be coming to our home to treat the child with their equipment such as the rollers, the wedges, the toys and things to make our children stand on….*”*(*female caregiver:mother)
A2: “…*What would be nice for us who wake up very early in the morning is to just wait at home and say that the physiotherapists are coming to help….”* (Female caregiver:mother)

Yet another caregiver observed and demanded;

A4: *“….We need more physiotherapists at clinics to manage the treatment of the children than always go to UTH….”*

The desire of one caregiver was expressed in this way;

A5: *“….We need teachers of disabled children also. We want our children also to be educated, be independent and improve their lives….”*

One caregiver expressed concern and disappointment on the government’s failure to provide assistance to the welfare of the cerebral palsy children and she said;

A1: *“….The government must start caring for these children, it is their responsibility to help us take care of their needs. They must help us, it is hard to provide for such a child…”*

Yet another caregiver expressed the following about government’s assistance to the children;

A3: *“….Why can’t the government bring more health workers here at CBIA clinic who are specialized in disabled children? It would……. be easier for us to bring our children nearer here for review at UTH than go to clinics all the time, it is far and expensive. The clinical officers here just give the child panadol when she is sick…”*

### Physiotherapy intervention satisfaction

The qualitative data revealed positive and negative responses from the patients in regard to satisfaction with physiotherapy services. The responses related to physiotherapy treatment sessions, type of treatment and involvement of caregivers and the results are also related to the findings on quantitative. Which revealed the following 60% (n = 15) of the caregivers were very satisfied (excellent) and those that were moderately satisfied (good) with the rehabilitation services offered to them were36% (n = 9) and those that responded that it was inadequate were 4% (n = 1).

A1: *“…There are so many weaknesses at the physio department, equipment is not enough and our children are attended to very late. There is an ‘I don’t care’ attitude towards clients…”* (Female caregiver:mother)
A3: *“…There are many children here at physiotherapy department but only few physiotherapists. Children are not given enough attention…“(*Female caregiver:sister)
A1: *“…Physios do very little at sessions because they are few…”* (Female caregiver:mother)
A2: *“…I don’t see what is done to the child because they never pay attention to me…”* (Female caregiver:mother)
A4: *“…I have a problem with my child being seen by different physiotherapists every week. One says this and the next week another one says something else. I need one physiotherapist to be seeing the patient all the time because inconsistency confuses me…”* (Female caregiver:mother)
A2: *“…Exercises only are done but there is no encouragement…*” (Female caregiver:mother)

The positive responses provided by the caregivers regarding physiotherapy were only related to the improvement of the child’s functional status.

A3: *“…Physiotherapy has brought a drastic improvement where the Childs’ functional ability is concerned. People should be informed about what physiotherapy can do….”* (Female caregiver:mother)
A4: *“…I am happy that the physios are doing a great job. The child couldn’t sit but now can. Considering her age we thought she would never sit. Am thanking the physios for making her sit…”* (Female caregiver:mother)
A5: *“…But since she started coming here (physiotherapy department) I have seen that she has improved…”* (Female caregiver:mother)

## Discussion

The study found that the median age of parents/caregivers was relatively lower (33.6 years; range 27 to 50 compared to 58.1, 56 and 58 median years found in other studies [16–18]. In some cases the median ages were higher; 65, 73, 75 and 80 years [19–22].

The results of this study reflected a dominant young age group being involved in cerebral palsy care-giving much less than any other age group in studies reviewed, however the findings in this study is very supportive of the Zambia’s scenario in accordance with the study by Eustis [15] who said that due to the issues of life expectancy dropping in Zambia in the past years, it is therefore expected that the care-giving role will be assumed mostly by the youths. This involvement has strong implications for young caregivers as they are in the productive age group in society but also issues of HIV and AIDS bring in already a lot of burden. According to Brehaut [23] parents and health-care providers need to pay attention to the effects of care-giving on selected areas of young peoples’ lives—particularly school and family life. They found that those who were of dating age, either did not date, or dated early to “get out of the home”. Concern has been mounting about the health and welfare of people who provide informal care for family or friends with cerebral palsy children. It is assumed that young and elderly people, who are vulnerable groups in their own right, may be carrying a particularly heavy burden [24].

The dominance of the younger age group assuming the responsibilities of caring for cerebral palsy patients may also be attributed to the age pattern in the country’s population, which has a dominantly higher middle-age group [25].

The MCSI [26] was used to measure the levels of strain and impact on the parents/caregivers. The results from this present study showed that the degree of strain as ascertained by the MSCI-13 was quite high in the caregivers who took part in this study with a p < 0.05. The results showed that the majority of the caregivers representing 64% had scores indicative of strain of a long-standing nature while only 36% had scores in the threshold of strain.

Further analysis of the MSCI-13 in the subscales detecting physical strain, financial strain, tiresome/inconveniencing and family changes showed significant and reliable differences with p < 0.05 (results not shown). On further discussion with parents/caregivers they further reviewed that they had stopped going for social functions such as kitchen parties or other social gathering because of social stigma. They further stated that sometimes they turned down the invitation because they were not comfortable leaving the child in the hands of someone they thought would not offer the care as they would. The interpretation is that the impact was translated into social, mental, medical and economic burden for the parent/caregiver.

The results of the present study were in agreement with the study done by Kaona and Tuba [27] which showed high psychiatric morbidity in parent/caregivers. The studies demonstrated that caring for a disabled child with CP had a negative impact on the parent/caregiver. Both the parent/caregiver needed support in the caring and upbringing of the child.

However, though the parents/caregivers were not admitting that the problem was there, when in actual fact the problem existed, was a source of concern. The study pointed to the fact that parents/caregivers were suppressing their emotions. The possible explanation to this situation could be attributed to traditional or cultural beliefs just as the current study indicates 92% of women, not admitting that the problem was there. Their responses were further confirmed from the answers the women were giving stating that the child was a gift from God. The suppression of the burden of care was demonstrated on the score on the MCSI subscales of physical strain. The results showed that all the women had signs of strain. The majority respondents had scores indicative of strain of long-standing.

Follow up tests on the effects of demographic variables on the MCSI Questionnaire was done by use of Multiple Regression Equations and the results showed that none of the independent variables was a predictor of the MSCI scores (results not shown). The results of the study demonstrated that despite females indicating the difficulties they encountered in the care of the child, they did not take it as a burden. The explanation from the females was that because the child was their own they had accepted the child as the gift from God. The females accepted the child in his or her state and enjoyed carrying out the duties despite the difficulties. For example, the mothers pointed out that they had difficulties in feeding but enjoyed the task because if the child ate enough then the child was okay and made them feel satisfied about their care of the child.

One mother/caregiver said;

*“She eats very slowly especially solid foods, and it means that I stop whatever I am doing to ensure that she feeds adequately. Other family members have little patience and sometimes do not manage to feed her…….. I do it myself to ensure she is okay……..I enjoy it though.”*

The results of the present study are in support of what has been noted to be a pointer to some more serious problems which female caregivers could be facing especially in most African countries [28]. Most researchers suggest that social factors determine the increase of minor psychiatric morbidity in women caring for children [29]. This study shows that additional care tasks widen further the difference between the sexes in psychiatric morbidity whether caring for a child with disabilities or not. Caring for a child with cerebral palsy has greater impact on the mental health of mothers.

Mothers of children with disabilities resulting from CP may assume more roles than mothers of non-disabled children. Hastings [30] further suggested that parents of children with special needs may have to assume unusual roles, such as developmental interventionalist and liaison among multiple health care workers and family members. The additional roles are not only time consuming, but also may be counterproductive because conflict may occur between the role of a parents and that of a teacher or therapist. However, Grant [31] found in an ethnographic study that parents of children with cerebral palsy did not have time, energy or confidence to carry out home programmes recommended by therapists.

Birth and early childhood rearing can have an adverse effect on maternal mental health even when there is no disability in the child [32, 33]. It is therefore imperative that adequate methods are used to isolate the impact of child care giving and other family commitments which are prone to affecting maternal mental health.

## Conclusion

The study raise important concerns in regard to inadequate research done in the area of CP caregiving. The study highlights the difficulties and amount of input needed to address cerebral palsy parents/caregivers’ experiences and needs. Physiotherapists working in communities and health institutions are intricately involved in CP habilitation and rehabilitation and should be attentive and supportive to the parents/caregivers. One of the greatest limitations of this study are the smaller sample size in the quantitative component, though to a smaller part it was complemented by the qualitative part.

## Abbreviations

CP: cerebral palsy
UTH: University Teaching Hospital
SPSS: statistical package for social scientists
WHO: World Health Organisation
